# The Genome of the Toluene-Degrading *Pseudomonas veronii* Strain 1YdBTEX2 and Its Differential Gene Expression in Contaminated Sand

**DOI:** 10.1371/journal.pone.0165850

**Published:** 2016-11-03

**Authors:** Marian Morales, Vladimir Sentchilo, Claire Bertelli, Andrea Komljenovic, Nadezda Kryuchkova-Mostacci, Audrey Bourdilloud, Burkhard Linke, Alexander Goesmann, Keith Harshman, Francisca Segers, Fabien Delapierre, Damien Fiorucci, Mathieu Seppey, Evgeniya Trofimenco, Pauline Berra, Athimed El Taher, Chloé Loiseau, Dejan Roggero, Madeleine Sulfiotti, Angela Etienne, Gustavo Ruiz Buendia, Loïc Pillard, Angelique Escoriza, Roxane Moritz, Cedric Schneider, Esteban Alfonso, Fatma Ben Jeddou, Oliver Selmoni, Gregory Resch, Gilbert Greub, Olivier Emery, Manupriyam Dubey, Trestan Pillonel, Marc Robinson-Rechavi, Jan Roelof van der Meer

**Affiliations:** 1 Department of Fundamental Microbiology, University of Lausanne, Lausanne, Switzerland; 2 Institute of Microbiology, University Hospital Center and University of Lausanne, Lausanne, Switzerland; 3 Department of Ecology and Evolution, University of Lausanne, Lausanne, Switzerland; 4 Master in Molecular Life Sciences, University of Lausanne, Lausanne, Switzerland; 5 Bioinformatics and Systems Biology, Justus-Liebig-University, Gießen, Germany; 6 Lausanne Genomic Technologies Facility, Center for Integrative Genomics, University of Lausanne, 1015 Lausanne, Switzerland; 7 SIB Swiss Institute for Bioinformatics, Lausanne, Switzerland; MJP Rohilkhand University, INDIA

## Abstract

The natural restoration of soils polluted by aromatic hydrocarbons such as benzene, toluene, ethylbenzene and *m-* and *p-*xylene (BTEX) may be accelerated by inoculation of specific biodegraders (bioaugmentation). Bioaugmentation mainly involves introducing bacteria that deploy their metabolic properties and adaptation potential to survive and propagate in the contaminated environment by degrading the pollutant. In order to better understand the adaptive response of cells during a transition to contaminated material, we analyzed here the genome and short-term (1 h) changes in genome-wide gene expression of the BTEX-degrading bacterium *Pseudomonas veronii* 1YdBTEX2 in non-sterile soil and liquid medium, both in presence or absence of toluene. We obtained a gapless genome sequence of *P*. *veronii* 1YdBTEX2 covering three individual replicons with a total size of 8 Mb, two of which are largely unrelated to current known bacterial replicons. One-hour exposure to toluene, both in soil and liquid, triggered massive transcription (up to 208-fold induction) of multiple gene clusters, such as toluene degradation pathway(s), chemotaxis and toluene efflux pumps. This clearly underlines their key role in the adaptive response to toluene. In comparison to liquid medium, cells in soil drastically changed expression of genes involved in membrane functioning (e.g., lipid composition, lipid metabolism, cell fatty acid synthesis), osmotic stress response (e.g., polyamine or trehalose synthesis, uptake of potassium) and putrescine metabolism, highlighting the immediate response mechanisms of *P*. *veronii* 1YdBTEX2 for successful establishment in polluted soil.

## Introduction

Contaminated soils pose an environmental and public health challenge for human societies. Pollution with crude oil or petrochemicals, such as jet fuels, solvents or polycyclic aromatic hydrocarbons is frequent and can lead to increased human exposure to carcinogens [1–3]. Pollution also leads to severe decrease in species diversity and loss of ecosystem functions [4–6]. Bacteria able to use aromatic compounds as sources of carbon and energy could potentially eliminate harmful organic contaminants and help reclaim land. Stimulation of *in-situ* community growth by landfarming or other bioremediation techniques can be an effective measure to speed up the degradation of organic contaminants in soil [5, 7, 8]. Alternatively, inoculation of specifically enriched communities or purified strains to contaminated sites has been proposed to enhance biodegradation rates (i.e., bioaugmentation). Bioaugmentation, however, is not common practice, because of inconsistent achievements [5, 9]. Accordingly, we and others have proposed that the lack of success of inoculation efforts may be due to our poor understanding of the factors determining invasion and establishment of non-native bacteria into existing communities [10–12] and in the real environment [8, 9, 13]. Therefore, in this study we determined the genome-wide gene expression changes in a bacterial strain relevant for bioaugmentation during a transition from typical laboratory cultivation to nonsterile contaminated sand. We chose to study the transition phase upon inoculation into contaminated sand, because, based on previous experience with other strains [11, 14, 15], we expected that the inoculant would display the highest expression changes and that we would see induction of the key genes involved in sand adaptation and contaminant degradation. It is clear, however, that cellular reactions during the transition phase are not necessarily equivalent to those during an actual bioaugmentation process.

The bacterial inoculant used for this study is *Pseudomonas veronii* 1YdBTEX2, a strain isolated from a jet-fuel contaminated soil (Hradcany, Czech Republic) [16] and capable of using benzene, toluene, ethylbenzene, *m-* and *p-*xylene (BTEX) as sole carbon and energy sources. Since the strain was an abundant member of the bacterial community in the contaminated site, we expected that it would be well-suited to survive toxicity of monoaromatic solvents, which can potentially be detrimental to bacterial cells [17] and adapt to a variety of contaminated soils. A draft genome sequence of *P*. *veronii* 1YdBTEX2 has been recently published in 63 scaffolds, a largely fragmented state [18], which makes genome-wide expression studies more difficult to interpret. Thus, we first obtained a full and gapless genome sequence of strain 1XdBTEX2 using long read PacBio SMRT sequencing. The genome was re-annotated using automated pipelines and manual curation. Subsequently, genome-wide expression patterns were studied after 1-hour exposure of exponentially growing cells of strain 1YdBTEX2 to either toluene or succinate, either in liquid suspension or in nonsterile sandy soil. Gene expression was quantified by RNA-sequencing using Illumina technology and mapping of reads onto the curated genome. PacBio sequencing, manual curation, gene expression experiments and preliminary RNA-sequencing analysis were performed within the context of a Master class [19]. Our study provides valuable insights into the cellular functions involved in the early adaptation response to organic solvents in sandy soil.

## Materials and Methods

### Isolation of DNA, sequencing and assembly of the *P*. *veronii* genome

*P*. *veronii* strain 1YdBTEX2 was cultured at 30°C with 180 rpm rotary shaking on 21C minimal medium (MM) [20] with 10 mM succinate. Cells were harvested in exponential phase at a culture turbidity of 0.15 at 600 nm by centrifugation of 10 ml culture for 6 min at 5000 x *g*, washed once with MM by 30 s vortexing and centrifugation as above. Total DNA was extracted using a Power Soil DNA Isolation kit (MoBio Laboratories, Carlsbad, CA, USA) and further concentrated by ethanol-sodium acetate precipitation and 75% ethanol washing. DNA pellets were briefly dried at room temperature, dissolved in sterile water after which the DNA quality was analyzed on a 2100 Electrophoresis Bioanalyzer Instrument (Agilent Technologies, Santa Clara, CA, USA). Subsequently, 7.5 μg of DNA was fragmented at 4100 rpm for 1 min with a Covaris g-TUBE device (Covaris Ltd, Brighton, UK) and 5 μg was used for preparing sequencing libraries (SMRTbell template prep kit 1.0, Pacific Biosciences, Menlo Park, CA, USA). DNA sequencing was performed on a PacBio RSII instrument (Pacific Biosciences) at the Lausanne Genomic Technologies Facility using three v3 SMRT™ Cells, P4-C2 chemistry and 180 min recording time. A total of 22,335 sequence reads with a mean length of 7,392 nt were *de novo* pre-assembled into 9 contigs using the Hierarchical Genome Assembly Process [21], yielding an average genome coverage of 73 times. Contigs were compared with each other and with an already existing assembly of the *P*. *veronii* 1YdBTEX2 genome (NCBI accession number AOUH01000001, 63 scaffolds) to detect possible sequence overlap, by using MAUVE [22]. Contigs were manually reassembled using Phrap and Consed [23] resulting in three final separate contigs. Ambiguous contig boundaries were further verified by PCR and manually corrected, when necessary.

### Annotation

The genome of *P*. *veronii* 1YdBTEX2 was annotated with the automated pipeline of GenDB [24] and using Prodigal for gene prediction [25]. Annotations were further selectively and manually refined using key word searches and by comparison to SWISSPROT/UNIPROT databases [26] using Basic Local Alignment Search Tool (BLAST) [27]. Functional pathways were interpreted using the Kyoto Encyclopedia of Genes and Genomes (KEGG) [28, 29]. The following processes and genome elements were specifically examined: aromatic compound degradation, putative transporters and efflux pumps, chemotaxis and flagella, anaerobic and aerobic respiration, transposable elements, secretion systems, toxin-antitoxin systems and heavy metal resistances. Putative prophages in the *P*. *veronii* genome were identified using PHAST [30], whereas putative genomic islands were predicted using Islandviewer [31] followed by manual searching of repetitive boundary sequences. The web-based tool TA finder [32, 33] was used to detect possible toxin-antitoxin systems. Protein allocations to Cluster of Orthologous Groups were predicted using the Integrated Microbial Genomes & Microbiomes facilities [34].

### Global transcriptional reactions of *P*. *veronii* to toluene and/or sand exposition

The genome-wide transcription response of *P*. *veronii* strain 1YdBTEX2 to toluene and/or sandy soil in comparison to liquid medium was analyzed using RNA-sequencing. Cells were inoculated (~10^7^ ml^–1^) from exponentially growing liquid batch cultures on 10 mM succinate, as follows: 100 ml culture was centrifuged for 10 min at 4000 x *g* to collect the cells. Cells were resuspended in 15 ml of MM with 0.5 mM succinate, to avoid them going into starvation phase during the 1 h assay. Aliquots of 100 μl cell suspension were immediately transferred to 15 ml polypropylene screw cap centrifuge tubes containing either 2 g of sand with toluene (Sa-To), or 2 ml of MM with 5 mM succinate (Li-Su) or with toluene (Li-To). Further aliquots were complemented with up to 5 mM succinate and added to 2 g of sand (Sa-Su). Sand was collected in Spring 2015 from a beach near Lake Geneva (°31.072’N, 6°34.733’E) and dried at room temperature for 7 days. Toluene was dosed through the vapor phase by pipetting 100 μl into a 1 ml micropipette tip, sealed on one end and inserted in the centrifuge tubes, which were closed during incubation. All treatments were started in quadruplicates and incubated for 1 h at 30°C at 160 rpm. Sand microcosms contained 4.8% gravimetric water content.

After 1 h, the cells were extracted by adding 5 ml of sterile saline solution (0.9% NaCl) to the tube and vortexing for 30 seconds. In the case of sandy microcosms, larger particles were allowed to sediment for a few seconds. Supernatants were filtered over 0.22 μm PVDF Durapore membrane filters (Merck Millipore AG) by vacuum suction. The filter with the cells was removed, folded to have the cell layer facing inside, and immediately frozen in liquid nitrogen. Frozen filters were then placed into a Bead tube (MOBIO RNA PowerSoil® Total RNA Isolation Kit, MoBio Laboratories) and stored at -80°C until RNA isolation.

### RNA isolation

RNA was extracted using the RNA PowerSoil Total RNA Isolation Kit (MoBio Laboratories). Frozen filters were removed from −80°C and coarsely crushed inside the tube with help of an RNAse-free forceps, before continuing the bead-beating protocol as recommended by the manufacturer (MoBio Laboratories). The RNA pellet was resuspended in a final volume of 20 μl of RNase-free water. Contaminating DNA was removed first by treatment with DNA-free Removal Kit (Ambion, ThermoFisher Scientific, Waltham, MA, USA), followed by TURBO DNase (Ambion). RNA was then purified using the RNeasy MinElute Cleanup kit (QIAGEN,Valencia, CA, USA).

RNA quantity and quality in the purified solutions was verified by reading 260 nm absorbance and the absorbance ratios at 260/280 nm and 260/230 nm, by using a NanoDrop spectrophotometer (ThermoFisher Scientific). RNA was migrated on an Agilent 2100 Bioanalyser (Agilent Technologies) to verify the presence of intact 16S and 23S rRNA. Genomic DNA contamination was checked by PCR using specific primers for a unique region in chromosome 2. If necessary, DNaseI treatment was repeated until no further DNA contamination was detected. RNA samples were then depleted from ribosomal rRNAs by using the Ribo-Zero rRNA Removal Kit Bacteria protocol (Epicentre, Madison, WI, USA). Subsequently, the RNA was reverse-transcribed, tagged on both ends, indexed and amplified by PCR using the ScriptSeq™ v2 Bacteria kit and ScriptSeq™ Index PCR primers set 1 (Epicentre). The resulting directional RNAseq libraries were sequenced using single 100-nt read chemistry on a Illumina HiSeq 2500 platform (Illumina, Inc., San Diego, USA) at the Lausanne Genomic Technologies Facility.

### Data analysis

Read mapping, sorting and formatting of raw reads was done with Bowtie2 [35] and Samtools [36], using the finalized gapless *P*. *veronii* 1YdBTEX2 genome sequence as reference. Mapped reads were counted with HTSeq [37], then further processed and analysed with edgeR [38]. Only reads counted more than once per million in at least three replicates were kept. After normalisation of the counts, differential gene expression between pairwise conditions was tested in a modified Fischer exact test (as implemented in edgeR). ANOVA (also as implemented within edgeR) was used to detect differential gene expression with interpretation groups “carbon source” (toluene or succinate) and “environment” (sand or liquid).

Genes were called significantly differentially expressed between two conditions at a false-discovery rate of <0.05 and a fold-change >2, and were subsequently interpreted by using Gene Ontology (GO) analysis [39]. GO terms of *P*. *veronii* genes were inferred using the program BLAST2GO [40]. The web-based bioinformatics tool GOEAST [41] was then used to analyse GO data sets of significantly differentially expressed genes in each pair-wise comparison, under the TopGO “Weight” algorithm [42].

A network for the predicted aromatic compound metabolism of *P*. *veronii* 1YdBTEX2 was manually created in Cytoscape (version 3.2.1) [43] with nodes representing metabolites and edges representing gene expression data.

### Gene marker exchange and nitrate respiration assay

In order to delete the *nar* genes, we amplified a 0.68 kb region upstream of *narL* (PVE_r1g2542) and a 0.8 kb region downstream of *narI* (PVE_r1g2549) using primers that introduce XmaI (5' ttttttttttCCCGGGatgctcacccatccccac 3') and EcoRI (5' ttttttttttGAATTCtcaaccggcttgatacccc 3'), or XbaI (5' ttttttttttTCTAGAggcacgttgaggttgtagatg 3'), and XmaI (5' ttttttttttCCCGGGgcaaagcatgctcagcc 3') sites, respectively. PCR products were size-verified by agarose gel electrophoresis, purified using a Nucleospin Gel and PCR cleanup kit (Macherey-Nagel AG, Oensingen, Switzerland), ligated into pGEM-T-Easy (Promega AG, Dübendorf, Switzerland) and transformed into *Escherichia coli* DH5alpha. Plasmids were purified using a Nucleospin Plasmid kit (Macherey-Nagel) and sequenced for verification, after which inserts were recovered by digestion with XbaI and XmaI (Promega), or with XmaI and EcoRI, and ligated with plasmid pJP5603 I-SceI v2 [44], pre-digested with XbaI and EcoRI. *E*. *coli* DH5alpha-lambda-pir transformants were recovered on nutrient agar plates with 50 μg ml^–1^ of kanamycin. A plasmid with the correct inserts was purified and retransformed into *E*. *coli* S17-1 lambda-pir, from which it was transferred into *P*. *veronii* 1YdBTEX2 by conjugation as described elsewhere [45]. *P*. *veronii* recombinants were selected on MM agar plates containing 50 μg ml^–1^ kanamycin with toluene vapor as carbon source, and verified by PCR for the appropriate integration of the construct. Plasmid pSW-2 harbouring the gene encoding the SceI restriction enzyme was delivered into one of the *P*. *veronii* recombinants by a second conjugation, selecting for transconjugants on MM plates with 10 mM succinate, 10 μg ml^–1^ of gentamicin and 1 mM *m*-toluate (to induce *SceI* expression). *P*. *veronii* clones resistant to gentamicin but sensitive to kanamycin were recovered and verified for deletion of the *nar* genes by PCR. One clone (*P*. *veronii* Δnar, strain 5239) was stored and tested for growth on nutrient broth (NB) medium in presence or absence of 1 g l^–1^ nitrate under aerobic or microaerophilic conditions. Aliquots of 0.1 ml stationary phase cultures of *P*. *veronii* 1YdBTEX2 or Δ*nar* grown on NB were transferred to 15 ml glass vials with screw-caps, containing either 7.5 ml or 15 ml of NB supplemented with 1 g l^–1^ potassium nitrate. The vials were incubated at 30°C for 16 h without shaking. Cell growth and the production of gas bubbles was inspected.

#### Database submission

The curated genome of *P*. *veronii* strain 1YdBTEX2 was deposited in the European Nucleotide Archive under bioproject number PRJEB11417. The RNA-seq reads were deposited in the NCBI Short Read Archive under accession number SRP077862.

## Results

### *P*. *veronii* 1YdBTEX2 genome features

**Fig 1:**
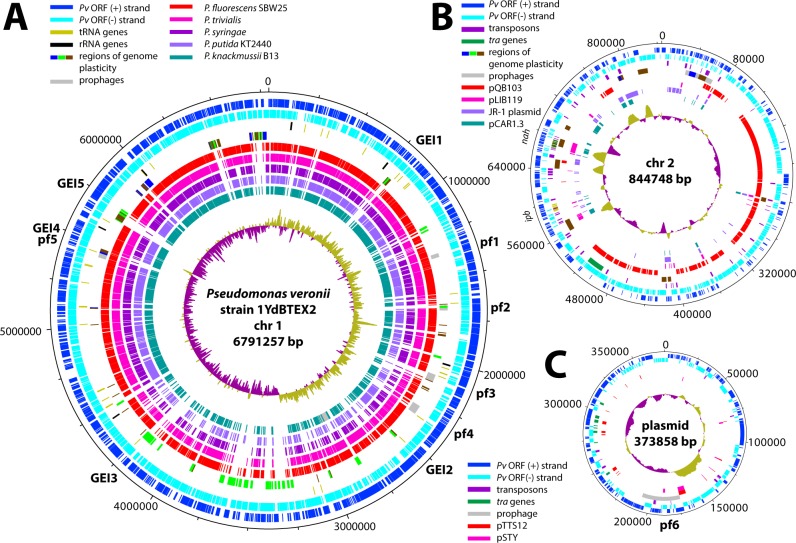
Circular maps of the replicons encompassing the *P*. *veronii* 1YdBTEX2 genome. (A) Chromosome 1 (chr1) with indication of possible genomic islands (GEI) and prophages (pf). The outermost circles show the location and orientation of predicted coding regions (blue and cyan), followed by tRNA (olive green) and rRNA genes (black), predicted regions of genome plasticity (blue-green-brown) islands and prophages (grey). The inner circles represent BLASTN comparisons with the close relatives *P*. *fluorescens* SBW25 (red, Acc. No. AM181176.4), *P*. *trivialis* strain IHBB745 (deep pink, CP011507.1), *P*. *syringae* pv. syringae B728a (dark purple, CP000075.1), *P*. *putida* KT2440 (light purple, AE015451.1) and *P*. *knackmussii* B13 (persian green, HG322950). GC skew (dark magenta and yellow green) is shown in the most central circle. (B) As A, but for the chromosome 2 replicon (chr2). Inner circles, from outwards to inwards, predicted transposons (dark purple) and *tra* genes (green), regions of genome plasticity (blue-green-brown) and prophages (grey), followed by BLASTN comparisons to *P*. *fluorescens* SBW25 plasmid pQB103 (red, AM235768.1, NC_009444.1), *Pseudomonas stutzeri* strain 19SMN4 plasmid pLIB119 (deep pink, CP007510.1), *Pseudomonas mandelii* JR-1 plasmid (dark purple, CP005961.1) and *Pseudomonas resinovorans* NBRC 106553 plasmid pCAR1.3 (Persian green, AP013069.1). (C) As B, but for the plasmid replicon. The inner circles represent the BLASTN comparisons with *P*. *putida* S12 plasmid pTTS12 (red, CP009975.1), and *Pseudomonas* sp. VLB120 plasmid pSTY (purple, CP003962.1). Plots generated with DNAPlotter [46].

**Fig 2:**
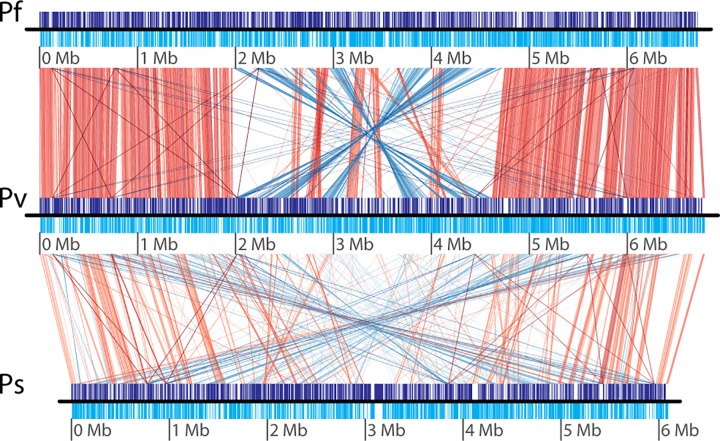
Comparison of the linearized *P*. *veronii* chromosome 1 replicon (Pv) to *P*. *fluorescens* SBW25 (Pf, Acc. No. AM181176.4) and *P*. *syringae* pv. syringae B728a (Ps, CP000075.1). Purple and cyan blocks show the location and orientation of coding regions on the positive and negative strands, respectively. Red and blue lines indicate direct and inverted colinear regions between both replicons, respectively, using a threshold of percentage nucleotide identity of 75%, maximum e-value for the region comparison of 1×10^−10^, and minimum overlap length for display of 1 kb. Plot generated with GenoPlotR [47].

Sequencing, assembly and manual refinement indicated that the genome of *P*. *veronii* strain 1YdBTEX2 consists of three replicons: a chromosome of 6,791,257 bp (named *chromosome 1*), a megaplasmid or second chromosome of 844,748 bp (named *chromosome 2*) and a plasmid of 373,858 bp (Fig 1). The plasmid has an extensive region of 23.5 kb (position 358719 spanning across to position 8288) highly similar to chromosome 1 (position 6746898 to 6770393, 99% identity). Chromosome 1 of *P*. *veronii* 1YdBTEX2 is closely related to *Pseudomonas fluorescens* SBW25 (AM181176.4), *Pseudomonas* sp. strain TKP (CP006852.1) and *Pseudomonas trivialis* IHBB745 (CP011507.1) (Fig 2, Figure A in S1 File), but aligns well with other Pseudomonas genomes such as *Pseudomonas syringae* pv. *syringae* B728a, *Pseudomonas putida* KT2440 or *Pseudomonas knackmussii* B13 (Fig 1A). In comparison to its closest chromosome relatives, *P*. *veronii* chromosome 1 has a large inversion of its central 2 Mb region (Fig 2, Figure A in S1 File). In contrast, there are no closely related sequences to chromosome 2 and the plasmid in public databases, although parts of other known plasmids can be detected (Fig 1B and 1C).

PHAST analysis identified 6 putative prophages in the *P*. *veronii* genome, five of which occur on chromosome 1 and one on the plasmid (Fig 1, *pf*, Table A in S2 File). Four of the prophages seemed intact and complete (pf1, 3, 4 and pf6, Fig 1) according to manual annotation and inspection. Putative prophage pf5 overlaps with a predicted genomic island (GEI4). IslandViewer results suggested 47 regions of genome plasticity (RGP) on chromosome 1 (Table B in S2 File). Five of those (GEI1-5, Fig 1A, Table B in S2 File) carry an integrase gene nearby or encompass a gene for a tRNA, and have a boundary repeat sequence, which are typical characteristics for a genomic island [48]. However, no genes encoding clear conjugation systems (e.g., type IV secretion system) could be identified within those five GEIs. Notably, there is a hypervariable region on chromosome 1 between position 3.2 and 3.8 Mb (Fig 1A), however, without characteristic gene markers for genomic islands. Eleven (chromosome 2) and one (plasmid) additional RGP were detected by IslandViewer on the other two replicons (Fig 1B and 1C). As these two replicons contain numerous genes encoding transposable elements or integrases (Fig 1B and 1C, Table B in S2 File), these may well have been implicated in creating the observed plasticity.

A total of 62.4% of genes on chromosome 1 could be assigned to clusters of orthologous groups (COGs), but only 24.2% and 30.0% of the genes on chromosome 2 and the plasmid, respectively (Figure B in S1 File), highlighting the large number of proteins of unknown function encoded in those two replicons. Compared to chromosome 1, chromosome 2 and the plasmid have a higher proportion of genes belonging to COG groups X (Mobilome: prophages and transposons), group L (Replication, recombination and repair) and M (plasmid, Cell wall, Figure B in S1 File). Chromosome 2 carries no genes for particularly obvious basic cellular functions, except two genes for sigma factors (PVE_r2g0923, *rpoS*; PVE_r2g0399, sigma-32), two genes for ribosomal proteins, and a second copy of the gene for DNA polymerase III beta-subunit (PVE_r2g0243).

**Fig 3:**
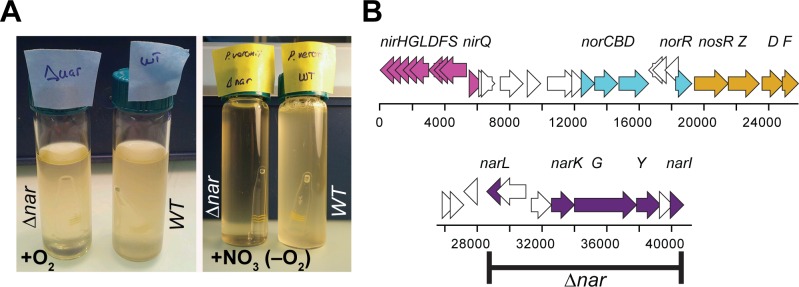
Overview of denitrification capacity of *P*. *veronii* 1YdBTEX2. (A) Overnight growth of *P*. *veronii* 1YdBTEX2 wild type (WT) and the Δ*nar* mutant in presence (+O_2_, left) or absence of air but with 15 mM nitrate supplemented medium (+NO_3_,–O_2_, right panel) conditions. Note the gas formation in the right panel of the WT incubation. (B) Gene regions predicted for denitrification in the *P*. *veronii* 1YdBTEX2 chromosome 1 with trivial gene names indicated. Black bar represents the deleted region in *P*. *veronii* Δ*nar*.

All genes required for aerobic respiration, such as NADH dehydrogenase, succinate dehydrogenase, cytochrome *c* oxidase, cytochrome *c* reductase, cytochrome *bd* complex, and an F-type ATPase were found encoded on chromosome 1 (Table C in S2 File). In addition, a superoperonic cluster encoding the enzymes required for a complete denitrification pathway (*nar*, *nir*, *nor* and *nos* genes, position 2644528 to 2685333) is located on chromosome 1, enabling the strain to grow under microaerophilic and anaerobic conditions with nitrate (Table D in S2 File, Fig 3A and 3B).

A complete set of genes for flagellar biosynthesis (e.g., *fli*, *flh* and *flg*) and classical chemotaxis (e.g., *cheA*, *Y*, *Z*, *W* and *B*) is encoded on chromosome 1 in a single large cluster (PVE_r1g4269-4321). Additional chemotaxis components are also encoded, which might be associated with swarming or twitching motility (e.g., PVE_r1g4595-4604 and PVE_r1g3912-3921, Table E in S2 File). *P*. *veronii* chromosome 1 further encodes a complete type I secretion system, a SecA-dependent type II system, putatively two type III systems, and three or four type VI secretion systems (Table F in S2 File). No complete type IV secretion system is encoded on chromosome 1, but both chromosome 2 and the plasmid carry genes encoding proteins with weak similarity to type IV secretion systems and may thus constitute atypical conjugative systems (Table F in S2 File). A large variety of putative toxin/antitoxin pairs were predicted by the web-based tool TA finder [33], 17 of which are found on chromosome 1, nine on chromosome 2 and two on the plasmid, covering known (e.g., *vapBC*, *higAB*, *relEB*, *hipAB*, *hicAB*) as well as less well-known families (Table G in S2 File).

Putative heavy metal resistances encoded by *P*. *veronii* include copper (*copRSCD*, *copAB*, both on chromosome 1, 2 and plasmid), mercury (*merRTPCA*, on chromosome 2), tellurite (*terZABCDE*, chromosome 2), chromium-zinc-cobalt (*czcRABCD*, chromosome 1), and arsenic (*arsRBCH*, twice on chromosome 1) (Table H in S2 File). Complete gene clusters for catabolism of 3-hydroxyphenylpropionate, toluene (see below), salicylate, phenol, anthranilate, vanillate and 4-hydroxybenzoate were detected in the genome of *P*. *veronii* 1YdBTEX2, scattered on all three replicons (Figure C in S1 File), allowing both *meta-* and *ortho-*cleavage of catechol intermediates. In contrast, no genes for benzoate 1,2-dioxygenase were found and *P*. *veronii* 1YdBTEX2 does not grow on benzoate.

### Analysis of the immediate catabolic response of *P*. *veronii* 1YdBTEX2 to toluene

**Fig 4:**
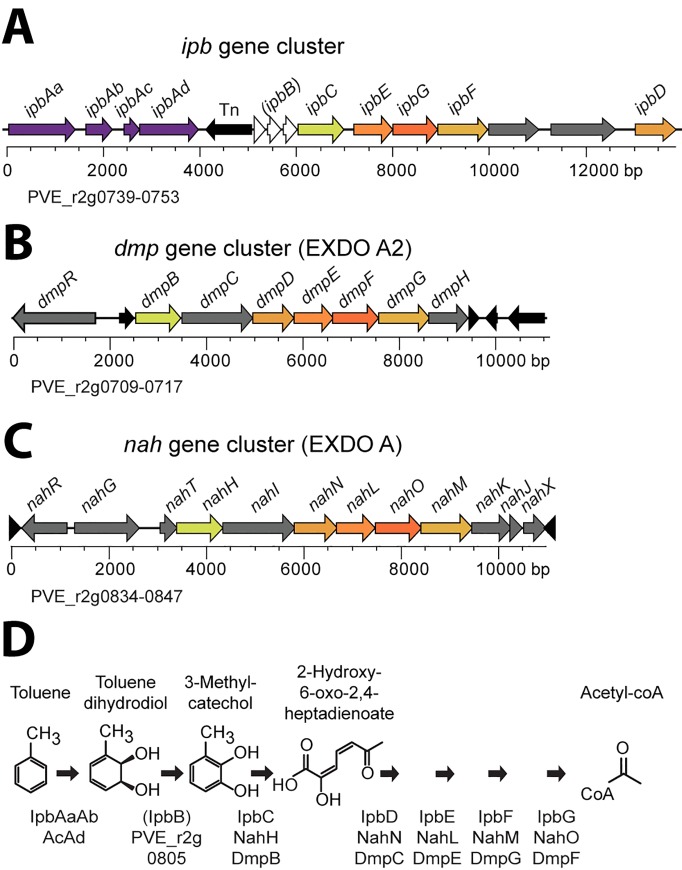
Organization of the clusters encoding genes involved in toluene degradation and catechol *meta-*cleavage pathways by *P*. *veronii* 1YdBTEX2. (A) Isopropylbenzene gene cluster (*ipb*). (B) *dmp*-like cluster (previously named EXDO A2). (C) *nah*-like *meta*-cleavage pathway genes (previously named EXDO A). Genes represented by arrows scaled to size (bp, base-pair) and relevant gene names as well as locus_tag (e.g., PVE_r2g0834) are indicated. Paralogous genes are colored similarly. Note the interruption in the *ipbB* reading frame by two stop codons (white arrows). Tn, transposable element (black arrows). (D) Proposed metabolic pathway for toluene to acetyl-CoA conversion with relevant chemical structures. Black arrows represent a single enzymatic step with the proposed involved (redundant) gene products indicated below.

*P*. *veronii* strain 1YdBTEX2 is mostly known for its capacity to degrade aromatic solvents such as BTEX [15, 16, 49]. Toluene and benzene are degraded through the same catabolic pathway but the enzymes potentially catalyzing these reactions are encoded in at least three to four gene clusters, several of which seem redundant [49]. Degradation of BTEX is thought to proceed via a multi-component aromatic ring dioxygenase, followed by a benzene-dihydrodiol dehydrogenase yielding catechol intermediates, and a *meta*-cleavage pathway, finally producing acetyl-CoA (Fig 4D) [49]. One of the involved gene clusters (*ipbAaAbAcAdBCEGFHD*) is localized on chromosome 2 (Fig 1B, Fig 4A), and includes both the genes for the aromatic ring dioxygenase (*ipbAaAbAcAd*) and a set of *meta-*cleavage pathway genes (*ipbCEGFHD*). However, the gene for benzene-dihydrodiol dehydrogenase (*ipbB*), is preceded by a transposon insertion and contains two non-sense mutations that interrupt correct translation (Fig 4A). The *P*. *veronii* 1YdBTEX2 genome encodes two further *meta-*cleavage pathways, both located on chromosome 2, within what seem to be a phenol degradation pathway *(dmpRBCDEFGH*, Fig 4B*)* and a salicylate catabolic pathway *(nahRGTHINLOMKJ*, Fig 4C). PVE_r2g0805, also located on chromosome 2, may be encoding an alternative benzene-dihydrodiol dehydrogenase (on average 56% amino acid similarity to the three *ipbB* protein fragments).

**Fig 5:**
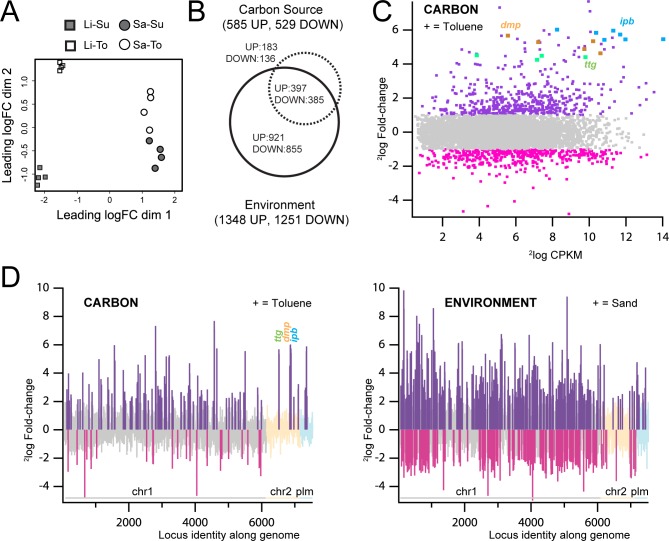
Genome-wide gene expression differences in *P*. *veronii* 1YdBTEX2 after 1 h exposure to different carbon sources or growth environment. (A) Two-dimensional Principal Component Analysis of quadruplate global RNA-sequencing data sets of *P*. *veronii* 1YdBTEX2 incubated in liquid medium with succinate (Li-Su), or toluene (Li-To), or in sand with succinate (Sa-Su) or with toluene (Sa-To). (B) Venn diagram with the number of unique and common genes significantly differentially expressed (2-way ANOVA, ^2^log-fold-change [logFC] >1, false-discovery rate [FDR] <0.05, *P* <0.01) as result of change of carbon source (succinate to toluene) or environment (liquid to sand). (C) Smear-plot of global gene expression intensity (^2^log CPKM) versus expression changes (^2^log fold change) compared between cells incubated with toluene (Li-To) versus succinate (Li-Su); in grey, genes not statistically differentially expressed (logFC<1, FDR>0.05, *P* >0.01); magenta, genes with lower, and dark purple, genes with higher expression in presence of toluene (+). Blue, *ipb* genes; yellow, *dmp* genes; green, *ttg* genes (toluene efflux pump). (D) Gene expression changes as an effect of carbon source (succinate versus toluene, left) or of environment (liquid versus sand, right), and plotted as function of genomic location (chromosome 1, chr1; chromosome 2, chr2 and plasmid, plm; organized according to locus_tag number). Bars indicate ^2^log-fold change. Dark purple, statistically significantly higher expressed genes in presence of toluene (+, left) or sand (+, right); cyan, lower expressed genes in pink. Positions of the *ipb*, *dmp* and *ttg* genes are highlighted.

To study which genes from those pathways are specifically induced upon exposure to toluene, which could indicate their implication in BTEX metabolism, a controlled RNA-seq transcriptome analysis was carried out. Four conditions were tested: cells incubated in liquid medium (Li) or in sand (Sa), and exposed to toluene (To) or to succinate (Su). RNA-seq analysis showed good replicate clustering for the liquid conditions and replicates grouped closely together in the Principal Component Analysis (both succinate and toluene, Fig 5A). A higher variability was observed among sand replicates, perhaps due to the higher heterogeneity of sand compared to liquid medium (Fig 5A).

Two-way ANOVA showed a total of 1114 genes with at least two-fold differential expression as a function of carbon source (false discovery rate < 0.05, *P* < 0.01), of which 319 (183 up and 136 down) were unique for carbon source and the remainder overlapping with the factor *environment*, (Fig 5B). About 50% of those encode conserved yet hypothetical proteins. The *ipbAaAbAcAd* genes, the *dmp* gene region and genes for a presumed BTEX efflux pump (*ttgGHI*, PVE_r2g0377-0383) showed among the highest observed transcript increase in presence of toluene (up to 54-fold, Fig 5C). The gene for C_4_-dicarboxylate transport (PVE_r1g4585) was among those whose expression decreased the most under exposure to toluene (down by 27-fold).

**Fig 6:**
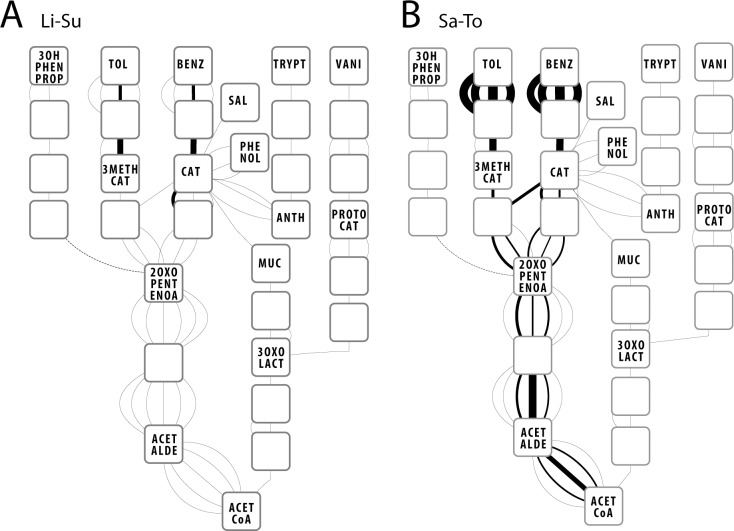
**Network analysis of gene transcription for deduced aromatic compound metabolism of *P*. *veronii* 1YdBTEX2 cultures in (A) liquid medium with succinate (Li-Su) and (B) sand with toluene (Sa-To).** Nodes represent substrates and metabolic intermediates. Edges represent the ^2^log-transformed average transcription across quadruplates (^2^log CPKM, counts per kilobasepair per million) of the gene coding for the particular enzyme that carries out the reaction between two nodes. Line width linearly decreasing between 100 and 1 for ^2^log CPKM between 50 and 5. ^2^log CPKM values lower than 5 all have a line width of 1. 2OXOPENTENOA; 2-oxopent-4-enoate, 3OHPHENPROP; 3-(3-hydroxyphenyl)propanoate, 3OXOLACT; 3-oxoadipate enol lactone, 3METHYCAT; 3-methylcatechol, ACETALDE; acetaldehyde, ACETCoA; acetyl-coA, ANTH; anthranilate, BENZ; benzene, CAT; catechol, MUC; cis,cis-muconate, PROTOCAT; protocatechuate, PHENOL; phenol, SAL; salicylate, TOL; toluene, TRYPT; L-tryptophan, VANI; vanillin. See Figure C in S1 File for details of all involved locus numbers.

To analyze the expression changes of *P*. *veronii* genes potentially implicated in aromatic compound metabolism more in general, we constructed a network with metabolites as nodes and known or predicted *P*. *veronii* gene functions as edges (Fig 6, Figure C in S1 File). Upon exposure to toluene there was a clear change in the expression level of the genes suspected to be implicated in BTEX metabolism, whereas expression of genes coding for other aromatic metabolic pathways remained unaltered (Fig 6, Figure D in S1 File). A clear exception was the gene for the putative alternative benzene-dihydrodiol dehydrogenase (PVE_r2g0805), whose expression was unaffected by the change from succinate to toluene (Fig 6, compare Li-Su with Sa-To). This gene (PVE_r2g0805) has previously been proposed to be recruited and to replace the interrupted *ipbB* gene within the *ipb* operon [18, 49, 50]. Multiple redundant genes encoding parts of *meta*-cleavage pathways for catechol metabolism are induced upon exposure to toluene. They are located on all three replicons (Fig 6, Figure C and D in S1 File) and one is part of a predicted prophage on the plasmid replicon (pf6, Table A in S2 File). No difference in the types of induced pathways was observed between liquid medium (Li-To) or sand.

**Fig 7:**
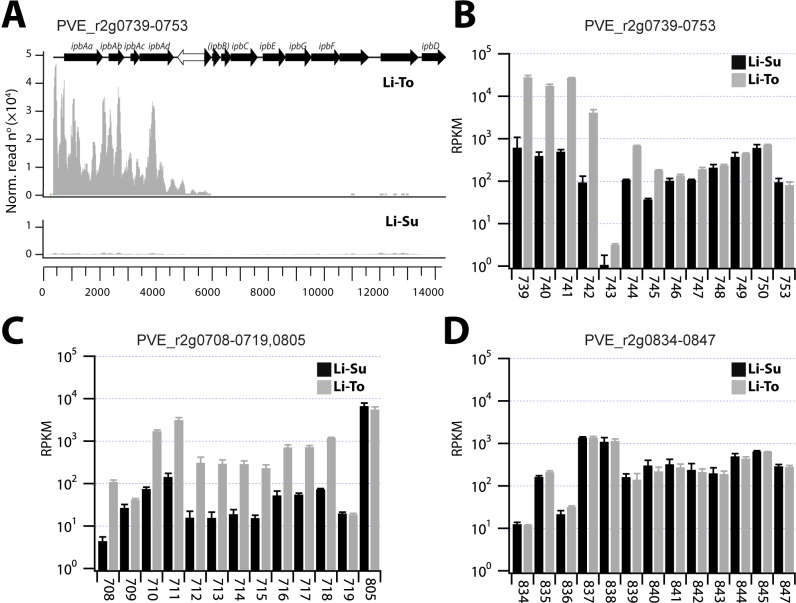
Comparison of catabolic gene transcription involved in toluene or *meta-*cleavage metabolism by *P*. *veronii* 1YdBTEX2 in liquid culture with succinate (Li-Su) or toluene (Li-To). (A) Normalized read counts across the *ipb* gene cluster (PVE_r2g0739-0753). Note the decrease as a result of the transposon insertion (white arrow). (B) Expression level (reads per kilobase per million, RPKM, ^10^log scale) of the *ipb* cluster genes (numbers refer to PVE_r2g loci). (C) as B, for the *dmp* cluster genes (PVE_r2g0708-0719), and the proposed gene encoding for the dihydrodiol dehydrogenase (PVE_r2g0805). (D) as B, for the *nah* cluster genes (PVE_r2g0834-0847).

By plotting the normalized read counts across the *ipb* gene cluster under conditions of liquid medium with succinate (Li-Su) or with toluene (Li-To), one can easily discern the premature transcriptional termination downstream of the *ipbAd* gene as a result of the transposon insertion (Fig 7A). Hence, whereas transcription of *ipbAa-Ad* is induced around 50-fold by exposure to toluene, there is basically no induction of the downstream genes for the *meta*-cleavage pathway (Fig 7B). In contrast, the genes in the *dmp* cluster were significantly induced by toluene in liquid medium (up to 20 times, Fig 7C), while transcription of those in the *nah* cluster remained unchanged (Fig 7D).

### Other functions affected by toluene exposure

As expected, GO analysis of genes whose expression is higher upon toluene exposure showed clear enrichment in the category *biological processes* for terms associated with aromatic compound metabolism (Tables I-K in S2 File). Terms associated with aerobic respiration, leucine biosynthesis and glyoxylate metabolism were also enriched during toluene exposure (Table K in S2 File). In contrast, for genes with decreased expression upon exposure to toluene, the following terms were enriched: cofactor and amino acid biosynthesis, energy reactions, reduced translation and post-translational reactions, as well as changes in the cell envelope. This suggests major cellular changes upon transition to toluene as growth substrate.

The expression of the gene PVE_r1g2050, which codes for a methyl-accepting chemotaxis protein (McpS), increased both in liquid and sandy microcosms upon induction with toluene (11.3 and 7.5 fold-change, respectively, Table J in S2 File). This may suggest that *P*. *veronii* has a similar chemotactic response towards aromatic solvents as *Pseudomonas putida* F1 [51]. Two chromosomally encoded toxin/anti-toxin systems (PVE_r1g1458/1459, PVE_r1g3189/3190) were also up-regulated upon induction with toluene (8-fold and 3-fold, respectively, Table G in S2 File). One of the predicted type VI regions (PVE_r1g5937-5956) showed a 2–4 fold lower expression in presence of toluene (Table F in S2 File).

### Analysis of the immediate response of *P*. *veronii* 1YdBTEX2 to change of environment

Gene expression differences as a function of growth environment (liquid culture versus sand) were far more dramatic than those as a function of carbon substrate. A total of 1348 genes (of which 921 unique) increased and 1251 genes (855 unique) decreased expression in cells exposed to sand compared to liquid medium (Fig 5B and 5D). GO analysis of enriched pathways suggests that cells in sand increase expression of osmoregulatory systems (e.g., alginate, glutamine, polyamine synthesis), and stress systems (e.g., oxidative stress, mercury defense, protein chaperones). They further redirect signaling and transcriptional regulation, produce reserve material (e.g., glycogen, fatty acid catabolism), and reprogram their central metabolism (Tables L-M in S2 File). A large variety of other genes are less expressed in cells added to sand compared to liquid, notably including those for RNA precursor synthesis and modification, respiration and cell shape (Table L in S2 File).

Several groups of genes putatively involved in osmoregulatory adaptation showed increased expression in sand, such as an *osmVWXY* osmoprotectant import system (PVE_r1g0889-0892), or transporters and synthesis of glycine-betaine (PVE_r1g0119,0120; PVE_r1g0416,0417, PVE_r1g5571-5576). Furthermore, two gene clusters associated with putrescine uptake (*potGHI* genes) and putrescine metabolism (*puuABGHF* PVE_r1g3330-3337) were up to 64-fold more expressed in sand (Table M in S2 File). These genes may code for a putrescine transamination system, leading to the osmoprotectant glutamate. In addition, all the genes coding for the KdpABC high-affinity potassium transport ATPase and its regulation (PVE_rg1744-1746) were up to 56-fold more expressed in sand (Table M in S2 File). This system is known to become expressed when turgor pressure is low [52, 53]. Finally, also genes putatively encoding trehalose biosynthesis (PVE_r1g2917,4895–4897) were higher expressed in sand (up to 42-fold, Table M in S2 File).

Other interesting individual genes or gene clusters whose expression changed significantly in sand include several for polysaccharide synthesis (PVE_r1g0180,0181; 1051–1055, 1533,1534; 3441–3446, Table M in S2 File), or for pili synthesis (PVE_r1g0867,0868; 1320, 4810). A long cluster of genes with encoded transporter systems of unclear specificity exhibited up to 35-fold higher expression in sand (PVE_r1g2142-2157, Table M in S2 File). Among the genes with the highest increase of expression in sand is a small cluster for hypothetical proteins (PVE_r1g0182-0184), with up to 650-fold higher expression. This suggests that these genes might play an important role in coping with adaptation to the soil environment. Several genes implicated in aromatic compound metabolism were also more expressed in sand (up to 10-fold), independently of toluene addition, suggesting the presence of other available aromatic growth substrates (e.g., PVE_r1g1095-1097; 1438–1446; 3731,3732, Table M in S2 File). We further identified higher expression levels of a cluster of genes in the plasmid involved in the resistance to copper (PVE_p0049-51, five-fold), of mercury resistance genes on chromosome 2 (PVE_r2g_0813–0817, threefold), and of chromosome 1 genes for a cobalt-zinc-cadmium efflux system (PVE_r1g06092-6099, up to 16-fold, Table H in S2 File). Part of prophage 2 (*pf2*) genes (PVE_r1g1579-1585) were up to 8-fold more expressed in sand, whereas many genes on *pf5* were less expressed in soil (Table A in S2 File). Additionally, three genes for an integrase showed increased expression in sand (PVE_r1g2409, twofold; PVE_p0204, p0207, 8-fold), suggesting that this condition can trigger movement of mobile DNA.

## Discussion

We determined a complete gapless genome sequence of *P*. *veronii* strain 1YdBTEX2, a wild-type BTEX degrader isolated from jet-fuel contaminated soil [16]. The genome measured a little over 8 Mb, which is almost 1.3 Mb larger than previously reported [18]. The genome appeared to consist of three replicons, which is unusual for pseudomonads. Chromosome 1 is most closely related to *P*. *fluorescens*, *Pseudomonas* sp. TKP and *P*. *trivialis* (Fig 2, Figure A in S1 File), although it contains a large number of unique regions (RGPs 1–27). Among these are four likely intact and one partial prophages, and suspected genomic islands (Fig 1), which underscores the various gene rearrangements by the wild-type strain 1YdBTEX2 with potential adaptive benefit. *P*. *veronii* 1YdBTEX2 contains a full set of genes essential for denitrification (Fig 4). This may enable the strain to survive and/or grow more easily under microaerophilic conditions in water-saturated soil. The two other replicons of the *P*. *veronii* genome have very little similarity to other known bacterial replicons (Fig 1B and 1C), and a large proportion of their genes code for hypothetical proteins, conserved or not (Figure B in S1 File). Although not tested here, both chromosome 2 and plasmid replicons may be self-transferable, which we deduce from the presence of gene functions with weak similarity but detectable homology to known type IV secretion systems (Table F in S2 File).

*P*. *veronii* 1YdBTEX2 is not particularly versatile in aromatic compound degradation (Fig 6), but the known pathways for BTEX degradation are all encoded on the chromosome 2. The hypothesis that BTEX degradation proceeds via an initial dioxygenation reaction catalyzed by the *ipbAa-Ad* gene products (PVE_r2g0739-0742) is supported by their more than 50-fold induction upon exposure to toluene (Fig 7A and 7B). The gene for dihydrodiol dehydrogenase (*ipbB*) is inactivated by two non-sense mutations and is preceded by a transposon insertion (Fig 4A). The dihydrodiol dehydrogenase function in toluene degradation is most likely complemented by the constitutively expressed gene PVE_r2g0805 (Fig 7C) [49]. Curiously, three *a priori* redundant *meta-*cleavage pathways are encoded on chromosome 2, the first similar to an archetype *dmp* phenol pathway, the second part of the *ipb* pathway and the third similar to a pathway from naphthalene/salicylate degradation (Fig 4). We confirmed the previous observation [49] that induction of the *ipb meta-*cleavage pathway in presence of toluene is abolished as a result of transcription termination inside a transposon inserted upstream of *ipbB* (Fig 7A and 7B). In contrast, the genes for the *meta*-cleavage pathway within the *dmp-*like cluster (PVE_r2g0710-0718) are induced upon toluene exposure, whereas those of the *nah-*like cluster (PVE_r2g0835-847) are not (Fig 7C and 7D). Nonetheless, the genes in all three clusters may contribute to *meta-*cleavage of catechol intermediates, since their expression levels in presence of toluene are quite similar (Fig 7, between 10^2^ and 10^3^ RPKM).

It is likely that effective growth on toluene of *P*. *veronii* 1YdBTEX2 also depends on an efflux system, which is encoded on chromosome 2 as well (PVE_r2g0377-0383). This three-component efflux pump and its two-component regulators are homologous to the *ttgGHI* system of *Pseudomonas putida* DOT-T1E [17] and are strongly induced in presence of toluene (Fig 6). It is plausible that *P*. *veronii* is even able to use toluene as a chemo-attractant, given the 10-fold induction of the *mcpS* gene PVE_r1g2050 on chromosome 1 (Table M in S2 File). This signal might be coupled to the flagellar systems encoded on chromosome 1 (Table E in S2 File). Toluene also induced expression changes in a range of other genes affecting various processes (Table J in S2 File). The induction of two toxin-antitoxin systems by toluene was intriguing (PVE_r1g1458/1459, PVE_r1g3189/3190), and may reflect a more general stress type response. For example, the *higBA* locus in *Mycobacterium tuberculosis* is more expressed under heat shock, nutrient starvation, DNA damage and hypoxia, supporting the hypothesis that it is involved in survival under harmful conditions [54]. *Sphingomonas wittichii* strain RW1, a degrader of dibenzodioxins, also induces an (entericidin-family) toxin-antitoxin upon exposure to sand [11].

The transition from liquid medium to sand (at 4.8% gravimetric water content) caused a plethora of changes in genome-wide gene expression patterns of *P*. *veronii* (Fig 5D, Fig 6C). This is surprising given that cells were resuspended in the same medium to inoculate the sand and the liquid. Likely, the small volume of inoculated liquid (with cells) formed small water films around sand particles and filled small pores between sand particles, which may have changed the availability of oxygen to the cells. Indeed, expression of genes involved in aerobic respiration was significantly reduced in sand compared to liquid, which we interpret as a better provisioning of oxygen to the cells in sand. Although counterintuitive, this makes sense given the low overall water content of the sand (4.8%). Diminished gene expression for aerobic respiration in sand under the same conditions was also a hallmark of the initial reaction of *S*. *wittichii* RW1 upon inoculation to sand contaminated with dibenzofuran or salicylate [11]. Most likely, the inoculated *P*. *veronii* cells also react immediately to the availability of micronutrients, such as copper and zinc (upregulated expression of transport systems in sand), but also to toxic trace metals like mercury (induction of the mercury defense system in sand). No changes in gene expression of transporters for nitrogen, phosphorous or sulfur were found upon inoculation in sand, but we assume that sufficient nutrients had been provided with the medium.

To better understand the changes observed after inoculation into sand and the cell response to water stress, we compared our results with previous data on differential gene expression in *P*. *veronii* 1YdBTEX2 induced upon matric (by addition of polyethylene glycol) or solute stress (addition of NaCl) [15]. Of the 274 differentially expressed genes in *P*. *veronii* 1YdBTEX2 during 1 h contact under either solute or matric stress, 150 were also significantly differentially expressed after 1 h contact in sand (Table O in S2 File). However, this is only 6% of all genes expressed in sand with at least two-fold difference (and FDR<1·10^5^). Therefore, we conclude that gene expression programs in sand have little in common with reactions to solute and matric stress, and that those are not appropriate proxies for the former as was previously postulated [15]. However, certain reactions were quite similar between solute or matric stress and incubation in sand, notably those involving Table O in S2 File). In contrast to what we expected, genes implicated in flagella biosynthesis and motility that previously had been found as marker genes for response to sand and water stress [11, 15] were not significantly differentially expressed in *P*. *veronii* 1YdBTEX2 under the conditions used here (Table O in S2 File). The reason for decreasing flagellar gene expression was proposed to be a relay of cellular energy for stress defense rather than for flagellar production and maintenance [55]. Nevertheless, others have shown that the reduction of motility of pseudomonads in response to water stress is not a generalized bacterial response to water-limiting conditions [56].

The accumulation of the compatible solutes glutamate and the uptake of potassium, mediated by the induction of the *kdpABC* operon, seems to be among the strongest “osmoprotectant strategies” employed by *P*. *veronii* 1YdBTEX2 cells to balance osmotic differences caused by alteration in the solute potential of the extracellular environment upon inoculation in sand (despite cells being inoculated in the same medium). Glutamate accumulation has been reported in *Pseudomonas syringae* strains B728 and DC30000 after osmotic stress, and the *kdp* operons in *Salmonella typhimurium* and *E*. *coli* are also induced upon K^+^ limitation and by osmolarity stress or turgor loss [57–59]. In addition, previous similar inoculation experiments in contaminated sand with the dibenzofuran-degrading bacterium *S*. *wittichii* also indicated the prime importance of glutamate biosynthesis to compensate for osmolarity differences [15]. Accordingly, we attribute the increase in putrescine metabolism (which produces glutamate) to the same osmotic response of *P*. *veronii* in sand. Following a fast potassium influx that increases not only the intracellular osmolality, but also the number of positive charges within the cells, polyamine efflux may occur to compensate for excess ionic charges. This response would be in line with three classical physiological responses of *E*. *coli* to osmotic stress: polyamine transport and accumulation of glutamate and potassium [58]. Analysis of enriched GO terms also indicated higher expression of *P*. *veronii* 1YdBTEX2 genes for trehalose and polysaccharide biosynthesis such as alginate upon contact to sand. This is similar to stimulation of alginate production by high osmolality in other *Pseudomonas* species [57], and alginate production has been proposed as a compensatory osmoadaptation mechanism in *Pseudomonas syringae* B728a [59].

Our data support the conclusion that inoculation of cells into sand, even when they are provided with buffered standard medium, provokes a major reorganization in global gene expression patterns (one-third of all genes) involving numerous different cellular pathways (Table J in S2 File). This is similar to previous observations and conclusions using inoculations in sand with *S*. *wittichii* RW1 [11]. However, the types of cellular pathways seem to bear very little similarity and may thus be quite specific for different bacterial taxa. This can be seen from the actually very few common GO terms enriched in sand incubations with *P*. *veronii* 1YdBTEX2 and *S*. *wittichii* RW1, which are phylogenetically distant species (comparison in Table P in S2 File, individual *P*. *veronii* reaction in Table L in S2 File). However, the reactions of *P*. *veronii* 1YdBTEX2 common to *S*. *wittichii* RW1 in sand suggest that during the first contact hour to sand the strain is not actively growing but rather scavenges and readjusts its metabolism to the new environment, while inducing the pathways for toluene metabolism in its presence.

Transcriptomic data are not sufficient to ascertain the capacity of strains to survive inoculation transition periods and successfully colonize a new environment. Nevertheless, our results suggest that *P*. *veronii* 1YdBTEX2 adapts to a polluted environment (here: sand plus toluene) based on multiple adaptive functions encoded in its genome. Future studies should thus try to confirm that *P*. *veronii* 1YdBTEX2 can actually grow on BTEX upon inoculation in contaminated material, and characterize global gene expression during growth and survival. It would also be interesting to examine global gene expression in BTEX contaminated materials in which strain 1YdBTEX2 cannot grow, which could help to identify factors critical for its survival and activity in the field. We identified a variety of highly up-regulated genes coding for conserved hypothetical proteins, which would be interesting to investigate further to understand their role in adaptation. The general understanding of factors that contribute or enable better strain survival in non-sterile soils with native communities is still extremely limited, and cannot be suitably predicted from laboratory studies involving standardized growth conditions with chemical stresses, as we demonstrate here. Such knowledge, however, is important for current and future efforts in synthetic engineering of microbial communities.

## Supporting Information

S1 File Figure A. Comparison of the linearized *P*. *veronii* chromosome 1 replicon (Pv) with its close relatives *Pseudomonas* sp. TKP (Ptkp Acc. No. CP006852.1) and *P*. *trivialis* IHBB745 (Ptri, CP011507.1). Figure B. Prediction of Cluster of Orthologous Group classification of coding regions in the *P*. *veronii* 1YdBTEX2 genome distributed across the three replicons. Figure C. Inferred metabolic network for toluene and aromatic compound metabolism by *P*. *veronii* 1YdBTEX2, displayed using Cytoskape 3.3.0. Figure D. Network analysis of toluene and aromatic compound metabolism by *P*. *veronii* 1YdBTEX exposed in (A) liquid to toluene (Li-To) or in (B) sand to toluene (Sa-To).
(DOCX)

S2 File Table A. Putative intact and incomplete prophages found in the genome of P. veronii strain 1YdBTEX2 and their differential gene expression. Table B. Putative regions of genome plasticity and genomic islands, and their differential gene expression. Table C. Differential expression of genes implicated in aerobic respiration and energy generation. Table D. Differential expression of genes for denitrification. Table E. Differential expression of genes associated with flagella, pilus, or chemotaxis. Table F. Differential expression of genes associated with secretion systems. Table G. Differential expression of genes for putative toxin/anti-toxin systems. Table H. Differential expression of genes for putative heavy metal resistance mechanisms. Table I. Differential expression of genes for putative aromatic hydrocarbon compound metabolism. Table J. Differential expression of genes in the genome of P. veronii 1YdBTEX2. Table K. Enriched GO terms among the significantly differentially expressed genes as response to change of carbon source. Table L. Enriched GO terms among the significantly differentially expressed genes as response to change of environment. Table M. Sorted genes significantly higher expressed in sand than in liquid. Table N. Sorted genes significantly lower expressed in sand than in liquid. Table O. Comparison of differentially expressed genes in sand versus under matric or solute stress. Table P. Common GO terms enriched in sand between *P*. *veronii* 1YdBTEX2 and *Sphingomonas wittichii* RW1.
(XLSX)
