# Supplementary material for: The Genome of the Toluene-Degrading Pseudomonas veronii Strain 1YdBTEX2 and Its Differential Gene Expression in Contaminated Sand
Source: PLoS One. 2016 Nov 3;11(11):e0165850. doi: 10.1371/journal.pone.0165850 (PMC5094676; doi:10.1371/journal.pone.0165850)
Supplement: S1 File — Figure A. Comparison of the linearized P. veronii chromosome 1 replicon (Pv) with its close relatives Pseudomonas sp. TKP (Ptkp Acc. No. CP006852.1) and P. trivialis IHBB745 (Ptri, CP011507.1). Figure B. Prediction of Cluster of Orthologous Group classification of coding regions in the P. veronii 1YdBTEX2 genome distributed across the three replicons. Figure C. Inferred metabolic network for toluene and aromatic compound metabolism by P. veronii 1YdBTEX2, displayed using Cytoskape 3.3.0. Figure D. Network analysis of toluene and aromatic compound metabolism by P. veronii 1YdBTEX exposed in (A) liquid to toluene (Li-To) or in (B) sand to toluene (Sa-To). (DOCX) [file pone.0165850.s001.docx]

**S1 File**

**Supplementary Figures to**

The Genome of the Toluene-Degrading *Pseudomonas veronii* Strain 1YdBTEX2 and its Differential Gene Expression in Contaminated Sand

Marian Morales^1^, Vladimir Sentchilo^1^, Claire Bertelli^2,7^, Andrea Komljenovic^3,7^, Nadezda Kryuchkova-Mostacci^3,7^, Audrey Bourdilloud^4^, Burkhard Linke^5^, Alexander Goesmann^5^, Keith Harshman^6^, Francisca Segers^4^, Fabien Delapierre^4^, Damien Fiorucci^4^, Mathieu Seppey^4^, Evgeniya Trofimenco^4^, Pauline Berra^4^, Athimed El Taher^4^, Chloé Loiseau^4^, Dejan Roggero^4^, Madeleine Sulfiotti^4^, Angela Etienne^4^, Gustavo Ruiz Buendia^4^, Loïc Pillard^4^, Angelique Escoriza^4^, Roxane Moritz^4^, Cedric Schneider^4^, Esteban Alfonso^4^, Fatma Ben Jeddou^4^, Oliver Selmoni^4^, Gregory Resch^1^, Gilbert Greub^2^, Olivier Emery^1^, Manupriyam Dubey^1^, Trestan Pillonel^2^, Marc Robinson-Rechavi^3, 7^, Jan Roelof van der Meer^1*^

Departments of ^1^Fundamental Microbiology, ^3^Ecology and Evolution, ^4^Master in Molecular Life Sciences, ^6^Lausanne Genomic Technologies Facility, Center for Integrative Genomics, University of Lausanne, 1015 Lausanne, Switzerland

2) Institute of Microbiology, University Hospital Center and University of Lausanne, 1011 Lausanne, Switzerland.

5) Bioinformatics and Systems Biology, Justus-Liebig-University, 35392 Gießen, Germany.

7) SIB Swiss Institute for Bioinformatics, 1015 Lausanne, Switzerland.

*Corresponding author

Jan Roelof van der Meer

Department of Fundamental Microbiology

University of Lausanne

Bâtiment Biophore, Quartier UNIL-Sorge

1015 Lausanne

Email: janroelof.vandermeer@unil.ch

**Figure A.** Comparison of the linearized *P. veronii* chromosome 1 replicon (Pv) with its close relatives *Pseudomonas* sp. TKP (Ptkp Acc. No. CP006852.1) and *P. trivialis* IHBB745 (Ptri, CP011507.1). Purple blocks show the location and orientation of coding regions on the positive and negative strands. Red and blue lines indicate direct and inverted colinear regions between both replicons, respectively, using a threshold of percentage nucleotide identity of 75%, maximum e-value for the region comparison of 1×10^-10^, and minimum overlap length for display of 1 kb. Plot generated with GenoPlotR (1).

**
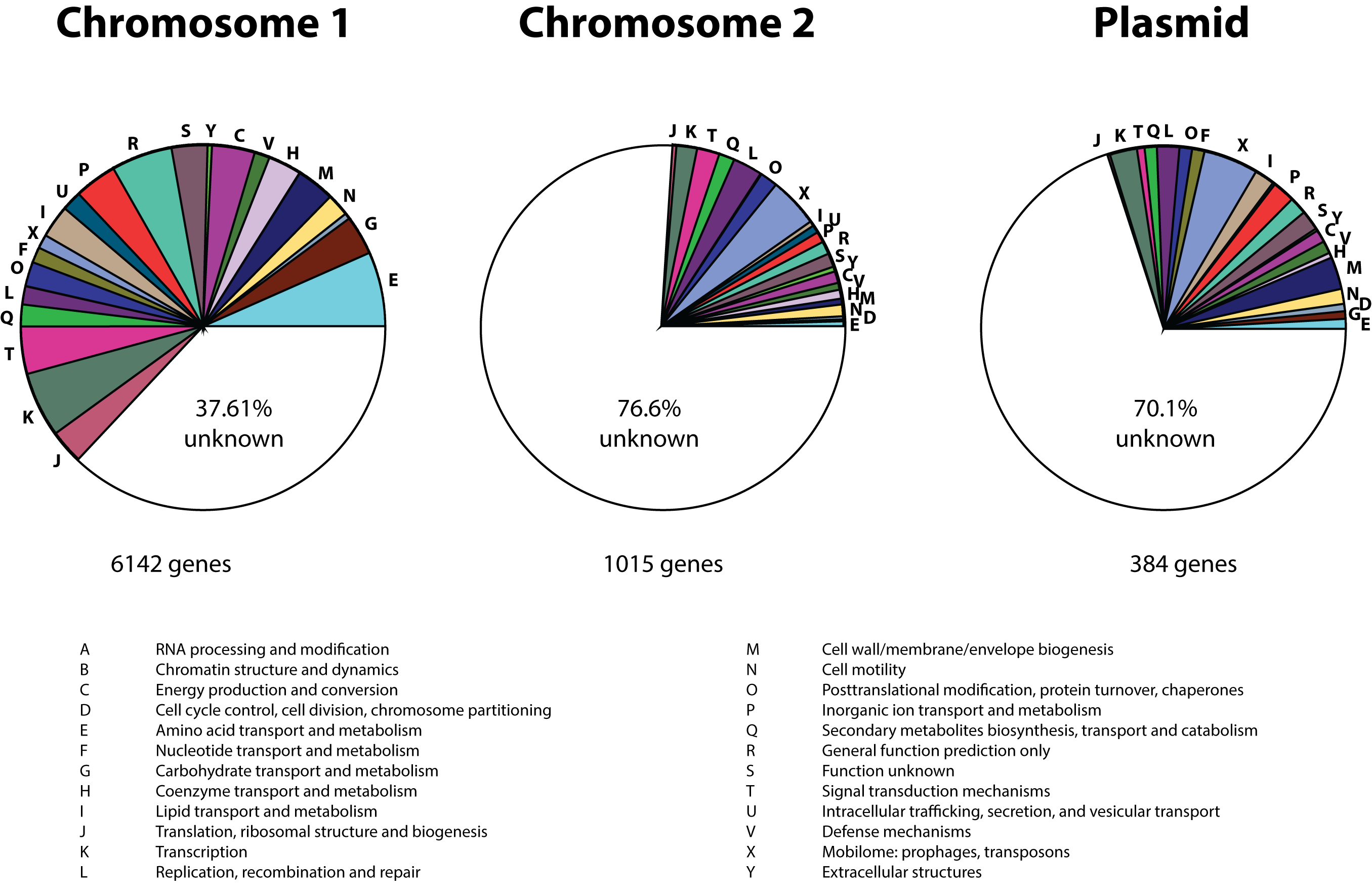
**

**Figure B.** Prediction of Cluster of Orthologous Group classification of coding regions in the *P. veronii* 1YdBTEX2 genome distributed across the three replicons. COG category distributions (A-Y) indicated as Pie-charts and specified below the diagrams. Total number of coding regions per replicon indicated. COG classification produced using the Integrated Microbial Genomes expert review system ([http://img.jgi.doe.gov/](http://img.jgi.doe.gov/er)).

**
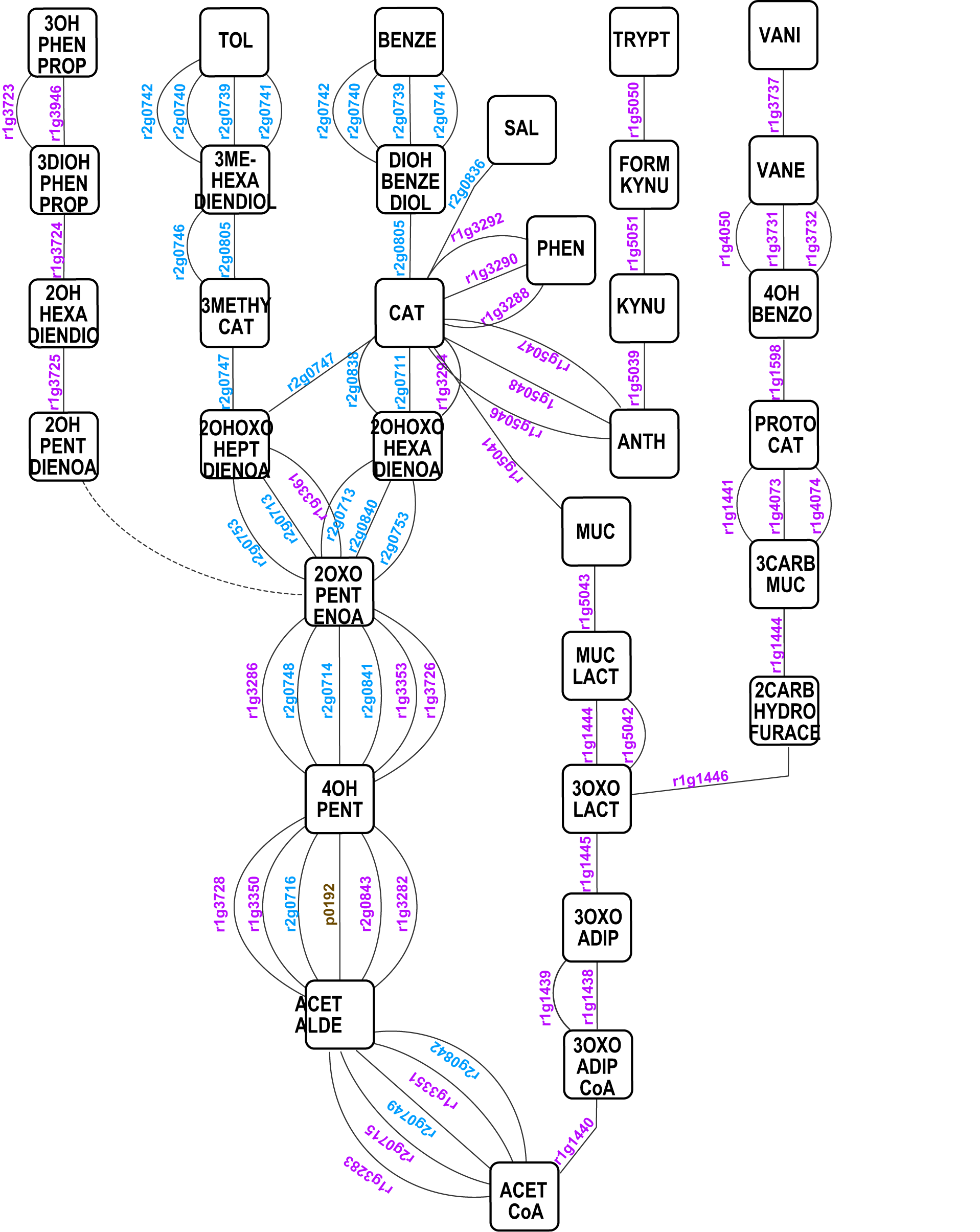
**

**Figure C.** Inferred metabolic network for toluene and aromatic compound metabolism by *P. veronii* 1YdBTEX2, displayed using Cytoskape 3.3.0. Nodes represent substrates and metabolic intermediates. Edges represent enzyme reactions converting the linked compounds. Numbers at the edges correspond to genes predicted to catalyze the indicated reaction. Cyan: genes on chromosome 1. Blue, genes on chromosome 2. Brown, gene on plasmid. Abbreviations**:**

2CARHYDROFURACE, 2-carboxy-5-oxo-2,5-dihydrofuran-2-acetate

2OHHEXADIENDIO, (2Z,4E)-2-hydroxy-6-oxonona-2,4-diene-1,9-dioate

2OHOXOHEPTDIENOA, cis,cis-2-hydroxy-6-oxohepta-2,4-dienoate

2OHOXOHEXADIENOA, (2Z,4E)-2-hydroxy-6-oxohexa-2,4-dienoate

2OHPENTDIENOA, (2Z)-2-hydroxypenta-2,4-dienoate

2OXOPENTENOA, 2-oxopent-4-enoate

3CARBMUC, 3-carboxy-cis,cis-muconate

3DIOHPHENPROP, 3-(2,3-dihydroxyphenyl)propanoate

3METHYCAT, 3-methylcatechol

3ME-HEXADIENDIOL, (1S,2R)-3-methylcyclohexa-3,5-diene-1,2-diol

3OHPHENPROP, 3-(3-hydroxyphenyl)propanoate

3OXOADIP, 3-Oxoadipate

3OXOADIPCoA, 3-oxoadipyl-CoA

3OXOLACT, 3-oxoadipate enol lactone

4OHBENZO, 4-hydroxybenzoate

4OHPENT, 4-hydroxy-2-oxopentanoate

ACETALDE, Acetaldehyde

ACETCoA, Acetyl-coA

ANTH, Anthranilate

BENZE, Benzene

CAT, Catechol

DIOHBENZEDIOL, cis-1,2-dihydrobenzene-1,2-diol

FORMKYNU, N-formylkynurenine

KYNU, L-kynurenine

MUC, cis,cis-muconate

MUCLACT, Muconolactone

PHEN, Phenol

PROTOCAT, Protocatechuate

SAL, Salicylate

TOL, Toluene

TRYPT, L-tryptophan

VANE, Vanillate

VANI, Vanillin

**
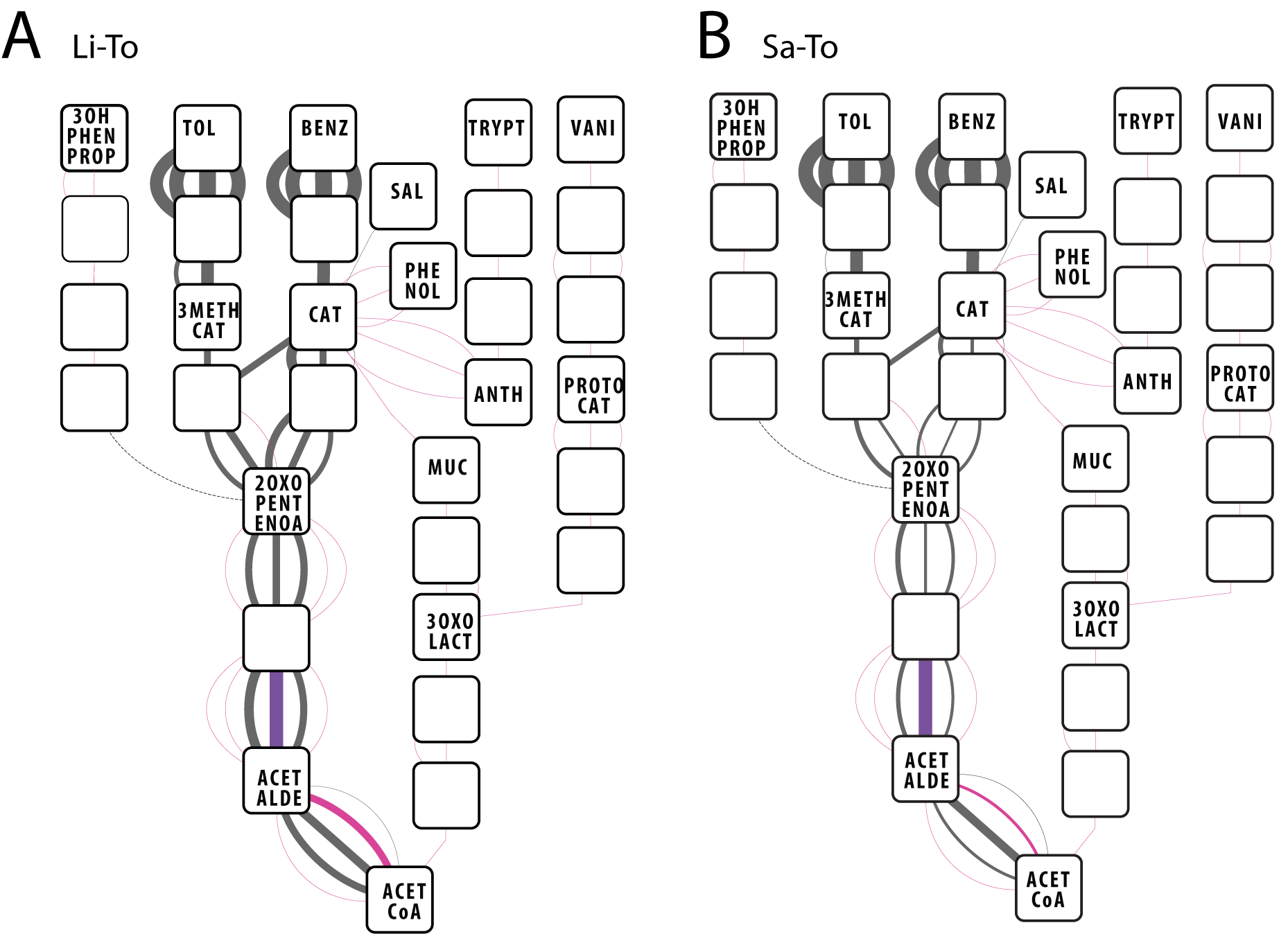
**

**Figure D.** Network analysis of toluene and aromatic compound metabolism by *P. veronii* 1YdBTEX exposed in liquid to toluene (Li-To, A) or in sand to toluene (Sa-To, B). Edge line thickness is a representation of normalized expression (2log CPKM) of the gene coding for the particular enzyme that carries out the reaction between two nodes. Normalization was carried out per gene among four conditions, taking the highest expression value as 100% (= line width 200). Abbreviations: 2OXOPENTENOA; 2-oxopent-4-enoate, 3OHPHENPROP; 3-(3-hydroxyphenyl)propanoate, 3OXOLACT; 3-oxoadipate enol lactone, 3METHYCAT; 3-methylcatechol, ACETALDE; acetaldehyde, ACETCoA; acetyl-coA, ANTH; anthranilate, BENZ; benzene, CAT; catechol, MUC; cis,cis-muconate, PROTOCAT; protocatechuate, PHENOL; phenol, SAL; salicylate, TOL; toluene, TRYPT; L-tryptophan, VANI;vanillin. Cyan lines, chromosome 1 functions; purple, plasmid function; grey lines, chromosome 2 functions.

**Reference**

1. **Guy L, Kultima JR, Andersson SG.** 2010. genoPlotR: comparative gene and genome visualization in R. Bioinformatics **26:**2334-2335.
